# Pyoperitoneum as a consequence of perinephric abscess spontaneous rupture. A case report

**DOI:** 10.1093/omcr/omad018

**Published:** 2023-04-20

**Authors:** Marouf Alhalabi, Rash Almokdad, Mouhammad Zaher Alhalabi, Mouhammad Alhalbouni

**Affiliations:** Gastroenterology Department, Syrian Board in Gastroenterology, Damascus Hospital, Damascus 35044, Syria; Gastroenterology Department, Syrian Board in Gastroenterology Damascus Hospital, Damascus 35044, Syria; Radiology department, Syrian Board in Radiology, Damascus Hospital, Damascus 35044, Syria; Surgery department, Syrian Board in Surgery, Damascus Hospital, Damascus 35044, Syria

## Abstract

Intra-abdominal infections are a common cause of severe sepsis and have a significantly high morbidity and mortality rate. Patients continue to present to hospitals with unacceptable delays in diagnosis or management, resulting in sepsis and organ failure, which lower their survival chances. We reported a rare case of a 64-year-old Syrian woman with a spontaneous rupture of a perinephric abscess that resulted in intra-abdominal infection and ascites, which led to sepsis and multiple organ failure despite resuscitation and antibiotic treatment according to guidelines. Although the recommendations for patients with intra-abdominal infection and hemodynamic instability differ, there is an agreement that surgery should be considered early when other interventional approaches have failed. Rupture of the perinephric abscess rarely produces intra-abdominal infection and ascites; effective care requires early and appropriate infection source identification. To avoid delays, doctors need to use academic methods in developing diagnoses and management.

## INTRODUCTION

Intra-abdominal infections (IAIs) are a common surgical emergency that has been linked to a significant increase in fatalities [1]. The most frequent surgical causes included acute appendicitis, acute calculous cholecystitis, acute cholangitis, diverticulitis, small bowel perforation and gastroduodenal ulcer perforation [1]. Treatment success depends on early and proper infection source identification, empirically broad-spectrum antibiotic coverage and rapid physiologic stabilisation with intravenous fluid therapy [2, 3]. Despite the significant advances in the availability and utilisation of laboratory tests and imaging, doctors continue to encounter challenges in the rapid diagnosis and treatment of IAIs. We reported a rare case of a 64-year-old woman who developed IAIs after a perinephric abscess spontaneous rupture consequent to ascites, severe sepsis and multiple organ failure.

## CASE REPORT

**Figure 1:**
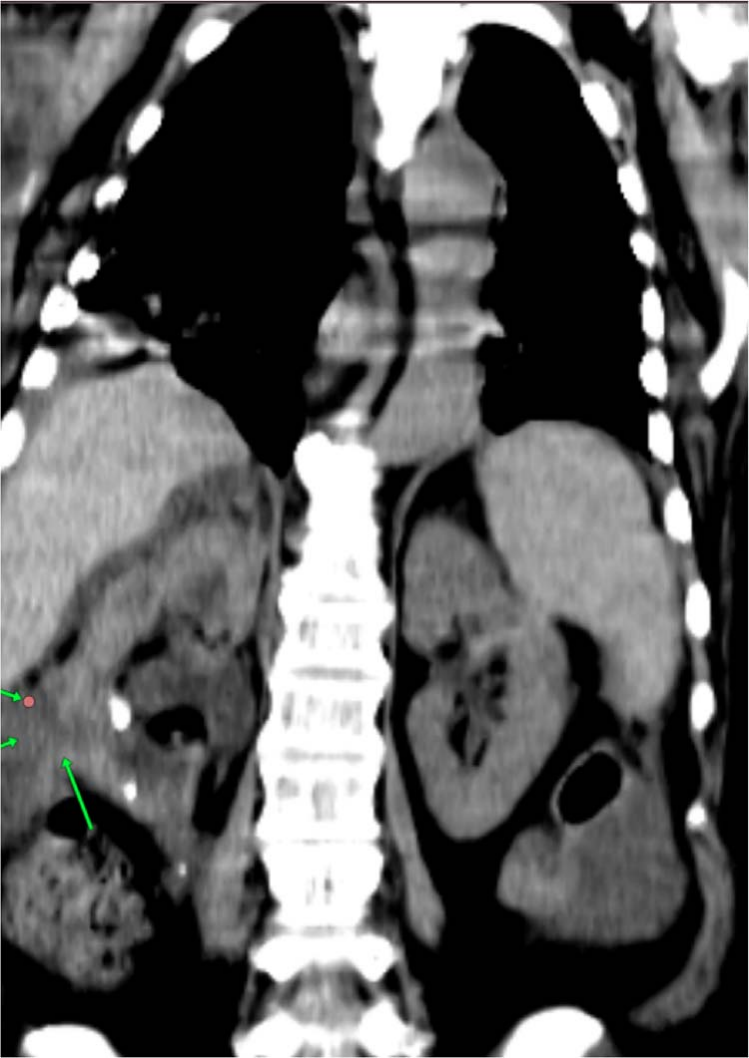
None enhanced computed tomography show kidney stones, ascites and swollen right kidney with the perinephric collection.

**Figure 2:**
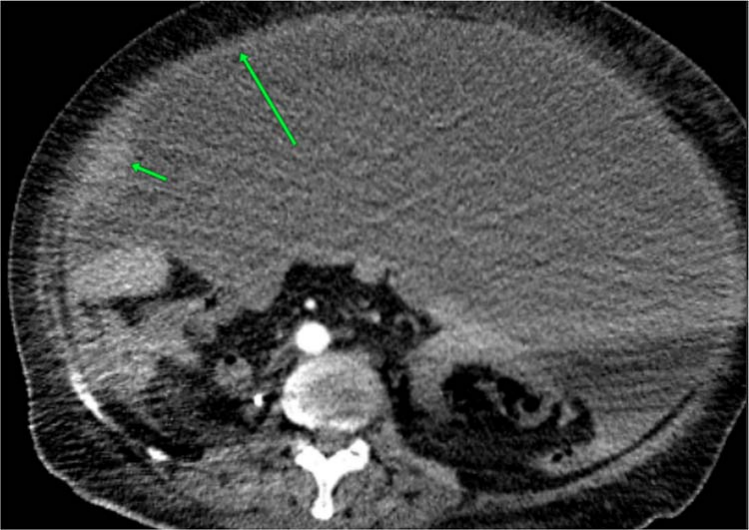
Enhanced computed tomography shows a large volume of ascites with grows and irregular peritoneum circumference.

A 64-year-old Syrian woman was referred for an ascites evaluation. She had been complaining of fever, abdominal pain and vomiting. Medical history included diabetes mellitus, hypertension, atrial fibrillation and a perinephric abscess diagnosed a week ago. The surgical history included laparoscopy and ureteral double-J stenting for a kidney stone one week earlier. Medications included bisoprolol 5 mg, apixaban 5 mg, metformin 500 mg, gliclazide 30 mg, ramipril 5 mg each once per day and ceftriaxone 1 g twice daily. Clinical examination revealed a surgical suture above the abdomen navel, an irregular heartbeat and a systolic murmur on the left sternum edge that increased with inhalation. There was shifting dullness, grade II extremities pitting edema and generalised abdominal tenderness without rigidity or muscle guarding. The peristaltic movements occur 4/minute. Her vital signs were as follows: Blood pressure = 90/50 millimeters of mercury, heart rate = 87/minute, temperature = 38°C and respiratory rate = 25/minute. One week prior, she had diagnostic ascites paracentesis, laboratory tests, and non-contrast-enhanced computed tomography (CT). Laboratory tests revealed urinary tract infection, acute kidney injury and secondary bacterial peritonitis (Tables 1 and 2). An abdominal non-contrast-enhanced (CT) scan discovered a perinephric abscess, kidney stones, ascites and a link between the kidney and the peritoneal cavity (Fig. 1). Doctors completed the workup with ascites cultures, adenosine deaminase activity to rule out tuberculosis, diagnostic laparoscopy and peritoneal biopsies that revealed suppurative inflammation. In the new admission, she had multiple organ failure evidenced by hypotension and increased creatinine level. She recorded two points on the SOFA and qSOFA scores [4] and was managed following standard resuscitation and hemodynamic support recommendations [2]. An abdominal X-ray showed a double J stent otherwise that was normal, as were laboratory tests including liver function tests and viral hepatitis serology. We completed the workup with diagnostic ascites paracentesis, and culture (Tables 1 and 2). The portal system and supra-hepatic vein ultrasound was normal, and the echocardiogram showed a normal ejection fraction of 65%. After 2 weeks, a contrast-enhanced multi-slice CT revealed ascites and an irregular peritoneum circumference (Fig. 2). Surgeons, through laparotomy drained multiple small abscesses and 6 L of pus. The pus collection was extended to the right perinephric space. There were no other intraperitoneal sources of IAIs detected. The fluid was sent for bacterial cultures. We continued with intravenous ceftriaxone 1 g twice, metronidazole 500 mg and vancomycin 500 three times a day, and she was discharged a week later.

**Table 1:** Patients’ tests

Test	First admission	Second admission	At discharge	Normal limits	Unit
WBC	10 900	7500	8500	4500–10 500	/mm^3^
N/L	68/28	60/30	66/20		
Hemoglobin	10	9.1	10	12–16	g/dl
Platelets	284	249	180	150–450	× 1000 mm^3^
Urea	55	66	31	15–54	mg/dl
Creatinine	1.3	2.1	0.5	0.5–1.3	mg/dl
GFR	41.2	23.7	124.2	90–120	mL/min/1.73 m^2^
Na	131	134	137	134–150	mmol/L
K	4.7	3.1	4	3.5–5	mmol/L
ESR	84	-			
CRP	137	98	19	Up to 6	mg/l
ALT	17	17		5–45	U/L
AST	16	10		8–40	U/L
Total bilirubin	0.5	-		0.5–1.2	mg/dl
Direct bilirubin	0.3	-		0–0.3	mg/dl
Total protein	5.1	6.1		6.2–8	g/dl
Albumin (blood)	2.4	2.8	-	3.8–5.1	g/dl
INR	1.9	2.3	1.3		
Glucose	129	147	139	74–106	mg/dl
Uric acid	7.2	-		2.5–6.5	mg/dl
ADA (serum)	12.1	-		Up to 17.7	U/L
ADA (ascites)	22.3	-		Up to 39	U/L
HBsAg		Negative			
Anti-HCV		Negative			

**Table 2:** Patients ascites tests

Test	First admission	Second admission	Range	Unit
	Result			
Albumin (serum)		1.2	3.2–5	g/dl
WBC (ascites)	15 100	8500		mm^3^
Neutro/lympho (ascites)	87/13%	85/15%		
Glucose (ascites)	10	25		mg/dl
LDH (ascites)		1350	75–160	U/L
Protein		2.26		
ADA	Negative	-	Up to 39	U/L
SAAG (Calculated)		1.48	-	-

SAAG: serum ascites albumin gradient is a formula used to assist in determining the etiology of ascites. SAAG = ascitic fluid albumin minus serum albumin.

## DISCUSSION

The infection caused by a perinephric abscess rupture is usually limited to the retro-peritoneum, and most cases are treated conservatively. Surgery is only required if additional pathology is suspected or if intraperitoneal structures are involved. IAIs are diagnosed through a detailed history, physical examination, laboratory studies and diagnostic imaging [3]. In rare situations, ascites may conceal the classic surgical abdomen by separating inflamed visceral and parietal peritoneal surfaces [5]. The diagnostic ascites paracentesis revealed a high serum ascites albumin gradient (SAAG) [6]. The differential diagnosis included liver cirrhosis and heart failure, which were ruled out by laboratory tests and radiological investigations [7]. Although spontaneous bacterial peritonitis (SBP) following portal vein hypertension is still possible, it is extremely rare, especially when secondary peritonitis is present according to the following criteria: absolute polymorphonuclear cell count>250 cells/mm^3^, glucose < 50 mg/dL and lactic dehydrogenase > upper limit of serum normal limits [6]. Both tuberculosis and malignancy were ruled out due to the high SAAG ascites [6]. The associated acute kidney injury in our case was part of a multi-organ failure resulting from severe sepsis, as liver cirrhosis is a requirement for the diagnosis of hepatorenal syndrome [8]. Despite the lack of evidence, we evaluated the treatment efficacy of IAIs by monitoring ascitic fluid neutrophil counts, the same as the evaluation of SBP treatment efficacy that should fall by at least 25% of the pre-treatment value [8]. The contrast-enhanced CT is a useful and precise method for detecting causative intra-abdominal infections and deciding the best intervention [1, 9]. The initial CT scan in our instance was non-contrast-enhanced, and although it provided useful information about kidney stones, kidney abscesses and ascites, it is not recommended for use in the case of intra-abdominal infections. It may miss diagnoses such as bowel perforation, adult appendicitis, complications from acute calculous cholecystitis and diverticulitis, as well as the fact that only contrast-enhanced CT can help to rule out other diseases such as ovarian pathology or aneurysms [1]. She had a GFR of 41.2 mL/min/1.73 m^2^, and there were concerns that the metformin would cause contrast-induced acute renal injury (metformin-associated lactic acidosis). The recommendations for metformin use differed. It is debatable whether metformin should be discontinued at the time of the contrast injection or 48 hours earlier, or whether it should be stopped for all patients or just those with underlying kidney disease [10]. The role of laparoscopy in the first admission should have been expanded to include draining abdominal fluid and looking for source control, whereas surgery in the second admission was postponed due to the patient’s hemodynamic instability. The recommendations for timing surgery in patients with IAIs and hemodynamic instability vary, depending on whether surgery should be performed immediately after being partially resuscitated [3], or whether surgery should be considered when other interventional approaches were inadequate [1, 2]. Early consideration of IAIs in the case of ascites next to a perinephric abscess may reduce sepsis, severe sepsis and multiple organ failure. Doctors need to use an academic methodology in developing diagnosis and management.
